# Linear Approaches to Intramolecular Förster Resonance Energy Transfer Probe Measurements for Quantitative Modeling

**DOI:** 10.1371/journal.pone.0027823

**Published:** 2011-11-16

**Authors:** Marc R. Birtwistle, Alexander von Kriegsheim, Katarzyna Kida, Juliane P. Schwarz, Kurt I. Anderson, Walter Kolch

**Affiliations:** 1 Systems Biology Ireland, University College Dublin, Belfield, Republic of Ireland; 2 Cancer Research Center, Georgia Health Sciences University, Augusta, Georgia, United States of America; 3 Beatson Institute for Cancer Research, Bearsden, Glasgow, United Kingdom; University of Hong Kong, Hong Kong

## Abstract

Numerous unimolecular, genetically-encoded Förster Resonance Energy Transfer (FRET) probes for monitoring biochemical activities in live cells have been developed over the past decade. As these probes allow for collection of high frequency, spatially resolved data on signaling events in live cells and tissues, they are an attractive technology for obtaining data to develop quantitative, mathematical models of spatiotemporal signaling dynamics. However, to be useful for such purposes the observed FRET from such probes should be related to a biological quantity of interest through a defined mathematical relationship, which is straightforward when this relationship is linear, and can be difficult otherwise. First, we show that only in rare circumstances is the observed FRET linearly proportional to a biochemical activity. Therefore in most cases FRET measurements should only be compared either to explicitly modeled probes or to concentrations of products of the biochemical activity, but not to activities themselves. Importantly, we find that FRET measured by standard intensity-based, ratiometric methods is inherently non-linear with respect to the fraction of probes undergoing FRET. Alternatively, we find that quantifying FRET either via (1) fluorescence lifetime imaging (FLIM) or (2) ratiometric methods where the donor emission intensity is divided by the directly-excited acceptor emission intensity (denoted *R_alt_*) is linear with respect to the fraction of probes undergoing FRET. This linearity property allows one to calculate the fraction of active probes based on the FRET measurement. Thus, our results suggest that either FLIM or ratiometric methods based on *R_alt_* are the preferred techniques for obtaining quantitative data from FRET probe experiments for mathematical modeling purposes.

## Introduction

Over the past 10 years, the number of genetically encoded, Förster resonance energy transfer (FRET)-based sensors for monitoring various biochemical activities in live cells and real time has skyrocketed [1], [2], [3], [4], [5], [6], [7], [8]. Most of these probes are unimolecular and have a general structure where a sensing unit, which is conformationally responsive to a biochemical activity of interest, is sandwiched between “blue-shifted” and “red-shifted” fluorescent proteins (FPs) capable of FRET. Thus, changes in biochemical activities of interest change the distance between the FPs, leading to detectable changes in FRET. For quantitative modeling of biochemical processes, these probes offer huge advantages over other currently available technologies (which include for example western blotting, immunofluorescence, and flow cytometery): (i) quantities of interest can be assayed in living cells and tissues [9] and in real time; (ii) high frequency sampling is possible; (iii) three-dimensional spatial data can be obtained; and (iv) single-cell as opposed to population average responses are measured. Although these characteristics make the use of FRET probes attractive for quantitative modeling, it is largely unknown how such data might precisely be used for such purposes. Therefore, in this work we first explore to what model variables FRET data should be compared. Moreover, to be useful for quantitative modeling, general good modeling practice dictates that the measured FRET should be linearly proportional to a modeled biochemical quantity. This is of particular importance when calibration curves are difficult if not impossible to obtain, which is the case for most of these sensors with the exception of those for small molecules such as Ca^2++^ and cAMP. There are two main methods for measuring FRET: ratiometric and lifetime imaging. We, therefore, also investigate how linear and quantitative FRET data obtained by these two methods are.

## Materials and Methods

### Cloning

All bacterial protein expression vectors were based on the backbone pQLinkGD (Addgene, Cambridge, MA), which includes an N-terminal GST tag for purification and a gateway cassette [10]. The fluorescent protein mTFP1 was obtained from Allele Biotech (San Diego, CA), mVenus was obtained by PCR amplification from the nuclear version of EKAR (Addgene, Cambridge, MA), and Raichu-RhoA-1237X was kindly provided by T. Ng. To construct the Raichu-RhoA probe with mTFP1 and mVenus, multi-site, three-way gateway cloning was used according to the manufacturer's protocol (Invitrogen), with mTFP1 in Position 1, the Raichu-RhoA sensing region in Position 2, and mVenus in Position 3. The Raichu-RhoA sensing region was defined as that being amplified by the forward primer 5′- TGGTCCCTGCTAGAGCAGCTGGGCC-3′ and the reverse primer 5′- ACCAGATTTTTTCTTCCCACGTCTA-3′. To amplify mVenus out of EKAR, we first digested EKAR with EcoRI/BamHI to remove Cerulean, which has significant sequence homology with mVenus. In the tandem mTFP1-Venus construct, the amino acid linker between mTFP1 and Venus was TGAGGGGLG.

### Protein Expression and Purification

The BL-21 strain of *E. coli* was transformed with the appropriate plasmids. Single colonies were then expanded in a 50 mL culture overnight in LB containing the appropriate antibiotic. The 50 mL culture was then added to 2 L of fresh LB, and bacteria were allowed to grow to an optical density of approximately 0.6. Protein expression was induced with 1 mM IPTG for 4 hrs at 37°C. The bacteria were pelleted (5,000 g, 10 min) and lysed in ice-cold PBS containing 1% Triton X-100, 1 mM PMSF, and 2 mM EDTA with sonication (3×30 sec bursts) on ice. The lysate was cleared by centrifugation (4°C, 13,000 rpm, 15 min, SS-34 rotor) and incubated with glutathione-sepharose beads (GE-Healthcare) overnight at 4°C with gentle rotation. Beads were pelleted and washed in Mobicol Mini-Columns (MoBiTec, Göttingen, Germany) 3× with lysis buffer and 2× with PBS, all at 4°C. Proteins were eluted by incubating the beads with 6 mg/ml reduced glutathione in PBS, pH 8.0 in Mobicol Mini-Columns 2× for 30 minutes. Protein concentrations were measured by absorbance at 280 nm on a NanoDrop spectrophotometer (Thermo-Scientific, Hertfordshire, UK), using 6 mg/ml reduced glutathione in PBS, pH 8.0 as the blank.

### Preparation of Raichu-RhoA-GTP and GDP

Raichu-RhoA-GST-glutathione-sepharose beads were incubated for 30 min at 37°C with either GTPγS or GDPβS binding buffer (20 mM Hepes, pH 8.0, 100 mM NaCl, 1 mM DTT, 1 mM EDTA, 10 mM MgCl2, 10 µM GTPγS or 100 µM GDPβS-all from Sigma) in a spin column (MoBiTec, Göttingen, Germany). The beads were subsequently washed 3× with binding buffer void of GTPγS or GDPβS and eluted by incubating the beads 2×30 min with glutathione elution buffer (20 mM Hepes, pH 8.0, 100 mM NaCl, 1 mM DTT, 1 mM EDTA, 10 mM MgCl_2_, 6 mg/ml reduced glutathione) at 25°C, as described above.

### Ratiometric Imaging

Protein solutions were prepared in the indicated proportions, and then loaded into a black 96-well plate. A plate reader (SynergyMX, BioTek, Mason Technology, Dublin, Ireland) was then used to excite the samples with 436/20 nm light for the donor and FRET channel or 505/9 nm light for the acceptor channel and scan emission wavelengths from 480 (FRET and donor) or 525 (acceptor) nm to 565 nm at 5 nm intervals. The donor channel was defined as the sum of emission intensities from 480 nm to 500 nm, the acceptor channel was defined as the sum of emission intensities from 525 nm to 565 nm, and the nominal FRET channel was defined as the sum of emission intensities from 515 nm to 565 nm. To vary the degree of overlap, decreasing 5 nm wavelength emission intervals were added to the FRET channel, starting with 535 nm to 565 nm and going down to 480 nm to 565 nm.

### Fluorescence Lifetime Imaging

Fluorescence lifetime measurements were performed using a Lambert Instruments fluorescence attachment (Amsterdam, The Netherlands) on a Nikon Eclipse TE 2000-U microscope equipped with a 40× objective and a filter block consisting of a 436/20 excitation filter, a T455LP dichroic mirror, and a 480/40M emission filter. A modulated 445 nm LED was used as light source to measure FLIM-FRET by frequency domain methods. Fluorescein (10 µM in 0.1 M Tris-Cl, pH>10), which has a known lifetime of 4.0 ns, was used as reference standard. Fluorescence lifetime, τ, was analyzed using the LI-FLIM software (version 1.2.1; Lambert Instruments, Amsterdam, The Netherlands). All measurements correspond to the lifetime estimated via the phase shift. Lifetime values were calculated for a rectangular region of interest of at least 100 by 100 pixels.

## Results and Discussion

### Relating FRET Data to a Biochemical Quantity of Interest

**Figure 1:**
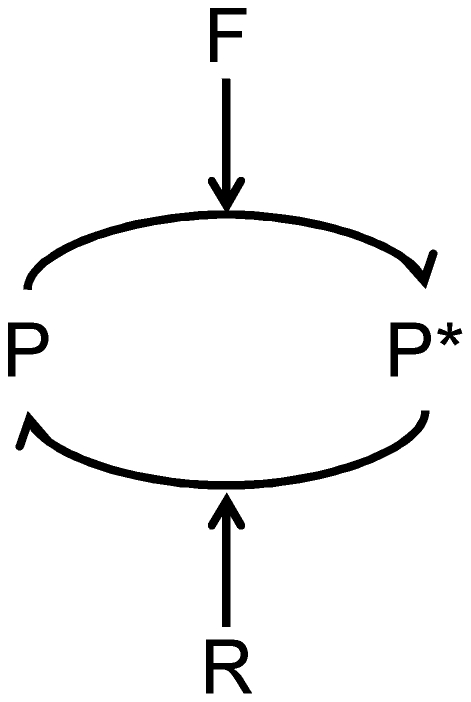
Kinetic Scheme for FRET Probe Activation. A forward enzyme F catalyzes the conversion of the FRET probe into an active state, and a reverse enzyme R catalyzes the conversion of the probe into an inactive state.

Before trying to understand the linear range of FRET measurements, it is important to understand precisely how the FRET signal is related to specific biochemical entities within the cell, such that FRET data can be compared to appropriate quantities in a mathematical model. In general, the transition of the inactive FRET probe P to an active state P*, where FRET is more likely to occur, can be represented by the scheme in Fig. 1. The enzyme F catalyzes the forward reaction and the enzyme R catalyzes the reverse reaction, both with a standard Michaelis-Menten mechanism. The fraction of donor fluorophores that are capable of transferring energy by FRET (*ϕ_F_*), is directly proportional to the concentration of active FRET probes, P* (the quantity *ϕ_F_* is related but not exactly equal to the traditional FRET efficiency *E*; this will be important and discussed in below analyses). Here, we consider how P* is related to biochemical quantities.

First, consider the scenario where the enzyme F is saturated (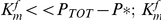
is the Michaelis constant for the forward enzyme and *P_TOT_* is the total probe concentration) and the enzyme R is in its linear range (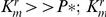 is the Michaelis constant for the reverse enzyme). Then, the steady-state level of P* is given by
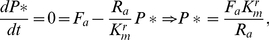
(1)where *F_a_* is the activity of the forward enzyme and *R_a_* is the activity of the reverse enzyme. Under these conditions, we see that the concentration of active probe molecules is linearly proportional to the forward enzyme activity. However, consider the steady-state levels of P* when R is saturated and F is in its linear range,
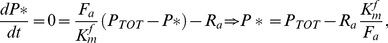
(2)or when both enzymes are in their linear range,
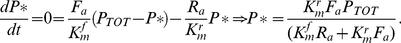
(3)


Equations 2 and 3 yield non-linear relationships between the forward enzyme activity *F_a_* and P*. Considering a more complex scenario where both enzymes follow Michaelis-Menten kinetics (not saturated nor in the linear range of operation) we have
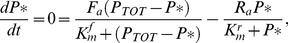
(4)which again gives a non-linear relationship between *F_a_* and P*. Considering non-steady-state or non-Michaelean conditions, which is particularly important given that intracellular reactions are rarely at steady-state or obey Michaelis-Menten kinetics, only further complicates the relationship between P* and forward enzyme activity. Therefore, we conclude that when FRET data are used for quantitative modeling, they should be compared to an explicitly modeled downstream substrate [11], [12], [13], [14], [15], [16], [17], [18], and not the activity state of the forward enzyme. For example, if the FRET probe is responsive to a kinase activity, then the amount of active FRET probes are proportional to the level of a phosphorylated substrate (which should be explicitly modeled), but not the kinase activity itself.

### Ratiometric Imaging

The most common way of quantifying FRET is by a technique called ratiometric imaging. The standard protocol in such an experiment is as follows [19]. Cells are exposed to excitation light for the donor channel, and then fluorescence emission is divided into donor and acceptor channels. The output from ratiometric imaging, *R*, is the intensity in the acceptor channel, *I_A_^don^*, divided by the intensity in the donor channel, *I_D_^don^*,
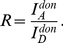
(5)


Below we derive an expression for R in terms of properties of intrinsic donor and acceptor properties, the excitation and emission channel characteristics, and of greatest importance, the fraction of donor molecules capable of transferring energy by FRET (*ϕ_F_*), which is directly indicative of the number of active probes.

The intensity in the donor channel is the sum of donor emission (*I_DD_*) and acceptor emission crosstalk (*I_DA_*),
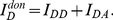
(6)


In most circumstances, it is reasonably easy to exclude acceptor emission from the donor channel, and therefore we assume that *I_DA_* is negligible compared to *I_DD_*. The donor emission can be represented in terms of the total number of excited donor molecules (N_D_^*^), the fraction of donor molecules actually transferring energy by FRET (the fraction of donor molecules capable of transferring energy by FRET multiplied by the FRET efficiency E of such molecules: *Eϕ_F_*), and the fraction of the donor emission captured by the donor channel (*f_DD_*), which has units of photons per molecule.
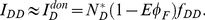
(7)


Note that *f_DD_* is proportional to the integral of the emission spectra between the emission filter wavelengths.

The intensity in the acceptor channel, similar to that of the donor channel, is the sum of acceptor emission (*I_AA_*) and donor emission crosstalk into the acceptor channel (*I_AD_*), 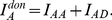
(8)


As above, the individual emission intensities can be represented in terms of the number of excited molecules, FRET fraction, and emission spectrum coverage, giving
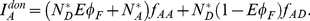
(9)


Here, the acceptor may be excited either by FRET from the donor (with the number of molecules denoted by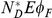) or by direct excitation at the donor wavelength (with the number of molecules denoted by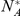), and *f_AA_* and *f_AD_* are the fractions of the acceptor and donor emission captured by the acceptor channel, respectively (again units of photons per molecule).

Given these expressions for the acceptor and donor emission intensities, the ratio R becomes
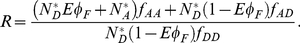
(10)


Providing that the amount of direct acceptor excitation is negligible, R simplifies to
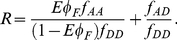
(11)


**Figure 2:**
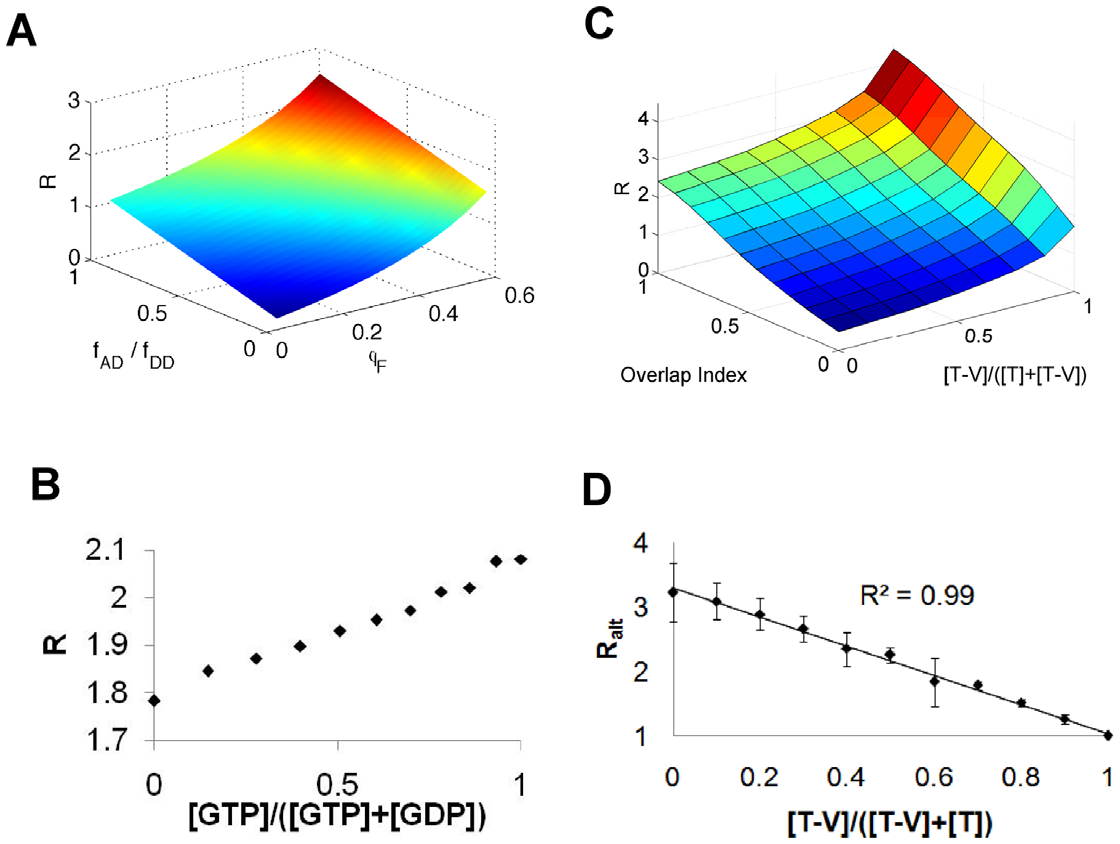
Ratiometric FRET. (**A**) Simulations for how the ratio R depends on the FRET efficiency *ϕ_F_* and the relative overlap of the donor fluorescence into the acceptor channel (*f_AD_*/*f_DD_*). The simulations were performed in MATLAB and are based on Eq. 11. Pseudo-color is indicative of z-axis (R) value. (**B**) Observed ratiometric FRET (y-axis) from various mixtures of GDP and GTP bound mTFP1-RaichuRhoA-mVenus. The proportion of GTP bound molecules is plotted on the x-axis. Data are representative of three independent experiments, and the observed ratio is based on the standard FRET channel (see Methods). Surface plots were not created to make the linear dependence clearer. (**C**) Observed ratiometric FRET from various mixtures of mTFP1 ([T]) and mTFP1-mVenus ([T-V]). The overlap index is a measure of how much mTFP1 fluorescence appears in the FRET channel (see Methods), and is proportional to the quantity *f_AD_/f_DD_* plotted in Panel A. Data are representative of three independent experiments. Pseudo-color is indicative of z-axis (R) value. (**D**) Observed ratiometric FRET, based on *R_alt_*, from various mixtures of mTFP1 ([T]), mTFP1-mVenus ([T-V]), and mVenus. Error bars denote standard error from 3 independent experiments.

The behavior of Eq. 11 is shown in Fig. 2A, for the conditions where (i) the apparent fraction of FRETing molecules (*Eϕ_F_*) ranges from 0 to a maximum of approximately 60% and (ii) the donor and acceptor emission coverages are of approximately the same magnitude (1∼*f_AA_/f_DD_*). Although condition (ii) may seem restrictive, in practice deviation from this condition can be easily adjusted at the level of digital intensities by changing detector gains for the acceptor and donor channels. A striking feature of Fig. 2A is the non-linearity of *R* as a function of *ϕ_F_* for all values of *f_AD_/f_DD_* (which quantifies the relative amount of crosstalk from the donor into the acceptor channel). Even with no crosstalk from the donor into the acceptor channel, ratiometric FRET should give a non-linear signal-response relationship.

To test this prediction of a non-linear relationship between *ϕ_F_* and the observed ratio *R* we analyzed a Raichu-RhoA FRET probe [7] that has mTFP1 [20] as the donor and mVenus as the acceptor [21]. This combination is superior to the previously used CFP/YFP pair as mTFP1 has a mono-exponential fluorescence decay, and is more photostable and brighter than ECFP (or its derivative mCerulean). The mTFP1/mVenus donor/acceptor combination has been shown previously to undergo significant FRET [22], [23]. The FRET output of this probe increases when it is bound to GTP, and decreases when it is bound to GDP. We expressed the Raichu-RhoA FRET probe in *E. coli* and purified it. To create active and inactive populations of the probe, we incubated the recombinant Raichu-RhoA protein *in vitro* with either GTPγS or GDPβS, which stably bind to small G-proteins such as those found in the Raichu-RhoA probe sensing unit, and therefore holds it either in the “on” (GTP) or “off” (GDP) state. We then mixed the GTP and GDP bound forms of the probe in various proportions and analyzed the ratiometric FRET with a fluorescence plate reader (Fig. 2B). To our surprise, the resulting relationship was linear across the entire spectrum of GTP bound fractions. However, the ratio R only ranges between approximately 1.8 and 2.1, which is quite small compared to the range calculated in Fig. 2A. Given this small ratio range, the inherently non-linear relationship predicted by Eq. 11 would appear effectively linear. Therefore, we tested a system where a greater range of ratios may be explored. We expressed and purified both mTFP1 alone (non-FRETing protein) and an mTFP1-mVenus tandem fusion (FRETing protein), mixed these two proteins together in various proportions, and then measured the resulting ratiometric FRET again with a fluorescence plate reader (Fig. 2C). With this system, we could explore a much wider range of ratio changes. The results confirm that the ratios depend non-linearly on the fraction of molecules undergoing FRET, in a manner consistent with the predictions of Fig. 2A. We verified that adding matched amounts of pure mVenus, rather than mTFP1-mVenus, did not change the observed ratios, showing that the observed ratio changes were a result of intra-molecular rather than inter-molecular FRET (data not shown).

An alternative method of quantifying FRET via ratiometric methods is to divide the donor emission intensity by the emission intensity of direct acceptor excitation (*I_A_^acc^*) [13], [14]. In this method, a reduction of the donor intensity is indicative of increased FRET, and this FRET indicator is normalized by the directly-excited acceptor intensity, which is an indicator of the total number of probes. In this formulation, one avoids introducing an inherent non-linearity into the denominator of the ratio. We denote this alternative ratio as *R_alt_*

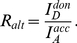
(12)


In the common situation where we can excite the acceptor without exciting the donor, and following the above nomenclature and assumptions, we arrive at
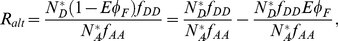
(13)which shows that the ratio *R_alt_* should be linearly proportional to the fraction of probes capable of FRET.

To test this hypothesis, we again mixed various dilutions of mTFP1-mVenus and mTFP1. But in contrast to the above experiment, as the concentration of mTFP1-mVenus was reduced, equal concentrations of both mTFP1 and mVenus were added to retain a constant total mVenus concentration (monomer plus tandem). The results confirmed that indeed there is a linear relationship between *R_alt_* and *ϕ_F_* (Fig. 2D, R ^2^ = 0.99). Note, however, that this same linearity result does not apply to *R_alt_* ^−1^, when one divides the emission intensity of direct acceptor excitation with the donor emission intensity.

### Fluorescence Lifetime Imaging

**Figure 3:**
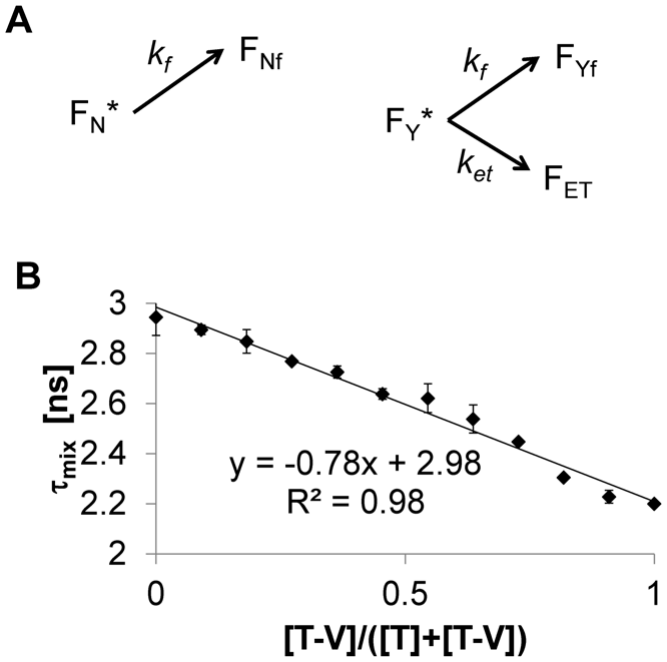
Fluorescence Lifetime Imaging. (**A**) Simple kinetic scheme for decay of excited FRET probes that are either capable (F_Y_^*^) or not capable of FRET (F_N_^*^). The molecules can relax by fluorescence (*k_f_*) or by FRET (*k_et_*). (**B**) Various proportions of mTFP1 and mTFP1-Venus were mixed in solution and their fluorescence lifetimes analyzed as described in Methods. The concentration of mTFP1 is denoted as [T] and mTFP1-Venus as [T-V]. We verified that adding corresponding concentrations of pure mVenus to decreasing amounts mTFP1 did not change the fluorescence lifetime of mTFP1, showing that this lifetime did not depend on mTFP1 concentration and that mTFP1 did not undergo significant intermolecular FRET given the concentrations used (0.1 mg/mL). Error bars denote 95% confidence intervals.

Another way of measuring FRET is by observing the lifetime of a population of excited donor molecules, which is commonly called fluorescence lifetime imaging microscopy (FLIM). When a donor molecule undergoes FRET rather than standard fluorescence emission, the average fluorescence lifetime decreases [24], [25]. In particular, the FRET efficiency *E* is related to the fluorescence lifetime by
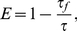
(14)where *τ* and *τ_f_* are the fluorescence lifetimes in the absence and presence of acceptor, respectively [25]. Now, consider a situation where there are two populations of excited donor molecules, one that is capable of FRET (F_Y_^*^) and one that is not (F_N_^*^), and FRET occurs with rate constant *k_et_* while fluorescence occurs with rate constant *k_f_* (Fig. 3A). Given (i) standard first-order kinetics for these processes and (ii) that the resultant measured lifetime of the mixture is a fractional weighted average of the individual components, it can be shown that 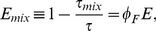
(15)where *E_mix_* and *τ_mix_* are the overall FRET efficiency and fluorescence lifetime of such a mixture of molecules, respectively. Condition (ii) will be the case so long as measurement error of the pure and FRETting lifetimes is the approximately the same, and the measurement method does not bias the estimate toward one population or another. Rearrangement of Eq. 15 gives
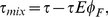
(16)which shows that the fraction of molecules capable of undergoing FRET, *ϕ_F_*, should be linearly proportional to the measured fluorescence lifetime *τ_mix_*. Moreover, both the slope and y-intercept of this line should be the related to donor fluorescence lifetime.

To test these hypotheses we again prepared mixtures of mTFP1 and the mTFP1-mVenus tandem fusion in various proportions, and then measured their fluorescence lifetimes (Fig. 3B). As predicted, we see a linear correlation between the fraction of mTFP1-mVenus molecules and the fluorescence lifetime (R^2^ = 0.98), with a y-intercept that agrees with that measured for pure mTFP1 (2.98 ns vs. 2.94+/−0.07 ns). Based on the calculated slope (−0.78 ns—see Fig. 3B) and the measured value of the mTFP1 lifetime (2.94 ns), we calculate the FRET efficiency of mTFP1-mVenus tandem molecules as 27% based on Eq. 16 (0.78/2.94). This corresponds closely to the measured FRET efficiency of 25% based on standard definition in Eq. 14 ((2.94 ns-2.2 ns)/2.94 ns), further justifying Eq. 16 and the calculated value of the slope.

### Relating Measured FRET to the Fraction of Active Probes

When comparing the measured FRET responses to a mathematical model simulation, it is useful to extract the absolute fraction of probes that are in the active state (ϕ_a_ = P*/P_TOT_). This is possible, so long as one uses either *τ_mix_* or *R_alt_* to quantify FRET (which both vary linearly with the fraction of probes capable of FRET), and can force all of the probes in a cell either to the active state or inactive state. If the probe is for a kinase, for example, then one can use saturating doses of phosphatase or kinase inhibitors to accomplish this. Then, the FRET measurements of these completely active and inactive states may be measured; denote these *F_act_* and *F_in_*, respectively. Here, FRET measurements may refer to either *τ_mix_* or *R_alt_.* Then, because the measurement scales linearly with the number of probes capable of FRET, the fraction of active probes can be written in terms of known quantities as follows
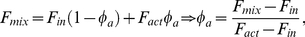
(17)where *F_mix_* denotes either *τ_mix_* or *R_alt_*. Thus, linear FRET measurements combined with simple controls allows for direct estimation of the fraction of active probes in the cell.

### Conclusions

Our theoretical analysis supported by experimental data yields important guidelines for using FRET probe data with quantitative modeling. We have shown that only in rare circumstances will the fraction of molecules capable of FRET be linearly related to an upstream enzymatic activity. Therefore, FRET data should only be directly compared to model variables analogous to the active FRET probe state, and not upstream enzyme activities. For instance, FRET data from the probe for ERK kinase, EKAR [2], should only be compared to the phosphorylation levels of an ERK substrate (corresponding to P*), and not the levels of active, doubly-phosphorylated ERK (proportional to enzyme activity). Of relevance to this study, FRET data from a probe for a small GTPase such as RhoA should only be compared to levels of RhoGTP (P*) or an explicitly modeled FRET probe, and not directly to GEF or GAP activities. Furthermore, we show that FRET measurements obtained via the standard ratiometric method have an inherently non-linear signal-response relationship and should therefore be avoided if possible. Although we found the dynamic range of the Raichu-RhoA FRET probe was not great enough to observe this inherent non-linearity, some probes have a greater dynamic range, and further probe improvements will push experiments into a regime where ratiometric measurements would become troublesome. Importantly, however, we find that measuring FRET either by (1) ratiometric methods where the donor emission intensity is divided by the emission intensity of direct acceptor excitation (*R_alt_*) or (2) FLIM (*τ_mix_*), results in a linear signal-response relationship between the measurement (*R_alt_* or *τ_mix_*) and the fraction of probes capable of undergoing FRET. Quantifying FRET via *R_alt_* removes the inherent non-linearity built into the denominator of the typically-used ratio *R*. As ratiometric methods involve less expensive, simpler microscope equipment than does FLIM, it may be preferable to use such methods, so long as data are quantified via *R_alt_*, and not with other commonly used forms of the ratio. Moreover, it has been shown in one case that ratiometric methods have a slightly better signal-to-noise ratio than lifetime imaging methods [2], albeit the difference is small, a non-linear form of the ratiometric method was employed, and it is not clear whether it holds true for different probes and fluorescent protein pairs. Also, some donors, such as ECFP, have multi-exponential lifetimes, and therefore are not suitable for FLIM. On the other hand, FLIM has several technical advantages. First, slight photobleaching of the donor will not affect FLIM measurements, whereas it would introduce significant artifacts into ratiometric methods, showing up as increased FRET. Second, photobleaching of the acceptor plays a minimal role in FLIM, but is likely to occur in ratiometric imaging due to direct acceptor excitation, and can introduce artifacts showing up as decreased FRET. This is particularly important given the well-known weak photostability of the YFP derivatives, which are commonly-used as acceptors. Lastly, dark acceptors, such as REACh [26], may be used in FLIM, greatly increasing the ability to multiplex FRET measurements for observing multiple activities in the same cell in real time. Thus, FLIM may be preferable when one is interested in analysis of networks over long time scales (or with high frequency measurements), which is usually the case in the context of mathematical modeling.
